# Suppression of *Arabidopsis* Mediator Subunit-Encoding *MED18* Confers Broad Resistance Against DNA and RNA Viruses While *MED25* Is Required for Virus Defense

**DOI:** 10.3389/fpls.2020.00162

**Published:** 2020-03-04

**Authors:** Nasser K. Hussein, Layla J. Sabr, Edina Lobo, James Booth, Emily Ariens, Swaminathan Detchanamurthy, Peer M. Schenk

**Affiliations:** ^1^ Plant-Microbe Interactions Laboratory, School of Agriculture and Food Sciences, the University of Queensland, Brisbane, QLD, Australia; ^2^ Plant Protection Department, College of Agriculture, University of Baghdad, Baghdad, Iraq

**Keywords:** *Alternanthera mosaic virus*, *Arabidopsis thaliana*, *Cauliflower mosaic virus*, *Cucumber mosaic virus*, mediator subunit, plant virus resistance, *Turnip mosaic virus*

## Abstract

Mediator subunits play key roles in numerous physiological pathways and developmental processes in plants. *Arabidopsis* Mediator subunits, MED18 and MED25, have previously been shown to modulate disease resistance against fungal and bacterial pathogens through their role in jasmonic acid (JA) signaling. In this study, *Arabidopsis* mutant plants of the two Mediator subunits, *med18* and *med25*, were tested against three ssRNA viruses and one dsDNA virus belonging to four different families: *Turnip mosaic virus* (TuMV), *Cauliflower mosaic virus* (CaMV), *Alternanthera mosaic virus* (AltMV), and *Cucumber mosaic virus* (CMV). Although both subunits are utilized in JA signaling, they occupy different positions (Head and Tail domain, respectively) in the Mediator complex and their absence affected virus infection differently. *Arabidopsis med18* plants displayed increased resistance to RNA viral infection and a trend against the DNA virus, while *med25* mutants displayed increased susceptibility to all viruses tested at 2 and 14 days post inoculations. Defense marker gene expression profiling of mock- and virus-inoculated plants showed that *med18* and *med25* mutants exhibited an upregulated SA pathway upon virus infection at 2 dpi for all viruses tested. JA signaling was also suppressed in *med18* plants after virus infection, independent of which virus infected the plants. The upregulation of SA signaling and suppression of JA signaling in *med18* may have led to more targeted oxidative burst and programmed cell death to control viruses. However, the susceptibility exhibited by *med25* mutants suggests that other factors, such as a weakened RNAi pathway, might play a role in the observed susceptibility. We conclude that MED18 and MED25 have clear and opposite effects on accumulation of plant viruses. MED18 is required for normal virus infection, while MED25 is important for defense against virus infection. Results from this study provide a better understanding of the role of Mediator subunits during plant-virus interactions, viral disease progression and strategies to develop virus resistant plants.

## Introduction

Resistance to infection in plants is often due to plant defense that limit multiplication or spread of a pathogen. As discussed by Klessig et al. (2000), some plants have the potential to identify pathogens and then activate a defense reaction when the products of resistance (R) gene expression interact with products of the virulence (Avr) gene expression. According to Revers and Nicaise (2014), the R genes that exist in the majority of the plant-pathogen integrations are part of the nucleotide binding site-leucine-rich repeat (NB-LRR).

Some types of plants possess mechanisms to detect plant pathogens by Pathogen-Associated Molecular Patterns (PAMPs). The main role of PAMPs is to act as the elicitors that permit plants to identify pathogens to mount the mechanisms that are ideally suited for their effective defense (Nürnberger and Lipka, 2005). PAMPs are advanced by plants through a different process known as Pattern Recognitive Receptors (PRRs) (Chiriac, 2013). This procedure involves the receptors that are triggered once the effect of pathogen proteins or other gene products is perceived by the plant. However, just as pointed out by Zvereva and Pooggin (2012), since there is currently no proof that PAMPs are linked with viruses at the moment, the primary defense mechanism for plants entail RNA muzzling, while the proteins are related to inducible resistance. Despite the fact that different viruses cannot be detected by PAMPs, the viruses still establish Pattern-Triggered Immunity (PTI) that makes it possible for the resistance (R) genes to identify various nonviral effectors together with the viral virulence proteins that are capable of producing Effector-Triggered Immunity (ETI). The procedure is similar to PTI even though it tends to be more intensive, since this type of immunity has the potential to activate different signals that will result in a hypersensitive reaction together with automated cell death (Nürnberger and Brunner, 2002). This reaction is an effort to locally respond to the virus by limiting its spread in the plants and needs triggering of the salicylic acid (SA) pathway that can result in systematic acquired resistance (SAR).

Defense signaling encompasses a methodical resistance that involves triggering cellular and the molecular defenses (Pieterse et al., 2009; Dong et al., 2015). Some plant pathogens are necrotrophs that require dead tissues for nutrients, while others are biotrophs that required live cells as hosts for completing their life cycles. In other cases, pathogens use plants for nectrotrophic and biotrophic life styles, but usually at different phases. The SA defense pathway typically confers resistance to biotrophic and the jasmonic acid (JA) pathway is typically effective against necrotrophic pathogens, with some exceptions (Pieterse et al., 2009). In many cases, however, it appears that virulent pathogens have the ability to trick the plant with effector molecules that induce the wrong pathway and as a consequence damage plants more (Thatcher et al., 2009). As viruses are biotrophs, an upregulation of the SA pathway would be essential for resistance, as this can result in localized oxidative burst by reactive oxygen species (ROS) production, hypersensitive response (HR), and programmed cell death, thus limiting the spread of viruses. Bacteria and fungi can feature biotrophic and nectrotrophic pathogenesis, while in the case of viruses, they are completely biotrophic pathogens. It is however important to take note that viruses are only able to reproduce intracellularly and can migrate and spread from cell-to-cell through plasmodesmata as well as over long distances using the vascular system, resulting in overall infection of their vulnerable hosts (Ellis et al., 2006). However before this occurs, certain pathogen-linked genes can trigger SAR which then activates resistance in plants whose distal tissues have not been affected (Zvereva and Pooggin, 2012).

The Mediator complex provides the link between transcription factors (TFs) and RNA Polymerase II, required for transcription. It contains approximately 31 and 34 subunits in mammals and plants, respectively (Mathur et al., 2011; Allen and Taatjes, 2015; Samanta and Thakur, 2015). The Mediator complex comprises three domains which are Head, Middle, and Tail, that, when taken together, are referred to as the core Mediator. In addition, a fourth module comprises Cyclin-Dependent Kinase 8 (CDK8) that is reversibly linked with Mediator. For yeast and metazoa, CDK8 controls transcription through phosphorylation of TFs and induction of RNA Pol II-Mediator interactions (Parker et al., 1996). The Tail domain of the Mediator complex interacts with the DNA-bound TFs, while the Head domain interacts with RNA Pol II and might also be engaged in either basal or activator-free transcription. For the Middle domain, Karijolich and Hampsey (Karijolich et al., 2012) discussed that it offers the flexibility needed by the huge protein complex so that it can show the necessary conformational changes in its reaction to RNA Pol II binding. The Mediator is capable of modulating RNA Pol II-based transcription by impacting the makeup of the preinitiation complex, Pol II stopping, elongation, and reinitiation and chromatic buildup (Allen and Taatjes, 2015).

MED18 is part of the Head domain of the Mediator complex where it can bind to TFs (Kim et al., 2011; Lai et al, 2014; Fallath et al., 2017). MED18 plays a major role in plant growth, flowering and immunity, including the production of noncoding RNA and modulates crosstalk between JA- and SA-associated defense pathways. Moreover, its *Arabidopsis* mutant *med18* plants show reduced miRNA levels and downregulated JA signaling and biosynthesis genes, while SA-associated PR and ROS producing genes are upregulated (Fallath et al., 2017). MED18 is believed to bind to MED20 in the Head domain of the Mediator complex and *med18* has similar growth phenotypes to *med20a* with strong resistance against the hemibiotrophic fungus *Fusarium oxysporum*, while showing increased susceptibility to necrotrophic fungi *Botrytis cinerea* and *Alternaria oxysporum*.

Contrary to MED18, MED25 is located at the Tail domain of the Mediator complex that connects to TFs such as MYC2 (Kidd et al., 2011). MED25 comprises 836 amino acids and is mechanically segmented into three different domains that are preserved (Bäckström et al., 2007; Kidd et al., 2009; Chen et al., 2012). The amino terminal of MED25 comprises of the domain Von Willebrand factor type A that is located in a preserved section of the Mediator attachment region. The subsequent domain comprises an activator interaction domain (ACID), that is vital for the interactions with TFs. Finally, the carboxyl terminal of MED25 comprises a poly track that is rich in glutamine at its C terminus, and most likely engaged in transcriptional triggering (Cerdán and Chory, 2003). MED25 plays a critical role in the growth of plants as well as response to stress. MED25 is reported to add to shoot elongation as well as flowering, based on the amount of the light that is present (Bäckström et al., 2007). Moreover, MED25 controls the adaptive nature of the plants perceived to avoid shade and MED25 is also needed for uncompromised expression of JA-dependent defense genes (Kidd et al., 2009; Elfving et al., 2011).

Common for both, *med18* and *med25* mutants is that they were found to be insensitive or partially insensitive, respectively, to JA (Kidd et al., 2009; Fallath et al, 2017). This defect in JA signaling for both plants is believed to mediate resistance to the hemibiotrophic fungal pathogen *F. oxysporum*, while being more susceptible to necrotrophic fungi *Botrytis cinerea* and *Alternaria brassicicola*. In this study, plant defense responses and resistance *Arabidopsis med18* and *med25* were tested against four viruses belonging to different families and compared to those in wild-type *Col*-0 plants. It was aimed to establish how MED18 and MED25 influence viral pathogenesis outcomes and whether there are common defense responses to different viruses (as was previously observed for fungal pathogens). In preliminary experiments, we found that *med18* mutants displayed resistance against *Turnip mosaic virus* (TuMV). Hence, we hypothesized that Mediator subunits may control common principles for broad resistance or susceptibility to different viruses, including TuMV, *Cauliflower mosaic virus* (CaMV), *Alternanthera mosaic virus* (AltMV), and *Cucumber mosaic virus* (CMV). All are ssRNA viruses apart from CaMV which is a dsDNA virus. This study can help to understand how plants interact with viruses and assist in establishing the pathways, including the roles of Mediator subunits MED18 and MED25, that were found to be required for normal virus infection and defense against viruses, respectively.

## Materials and Methods

### Cultivation of *Arabidopsis* Plants

*Arabidopsis med18* and *med25* homozygous mutant plants were previously characterized and maintained from our previous studies (Kidd et al., 2009; Fallath et al, 2017). The first stage included sowing wild-type *Col*-0, *med18*, and *med25 Arabidopsis thaliana* seeds and placing them at 4°C for 2 days. A growth chamber was used to cultivate seedlings under the following conditions: 8 h of 24°C during the day (160 μE m^-2^s^-1^) followed by 16 h at 21°C during the night. The last stage included transplanting the seedlings after 3 weeks (two plants per pot), and then inoculating them with viruses when they were 5 weeks old. All plants were grown in parallel [including mock-inoculated-/virus-infected plants for each genotype and both time points of sampling (2 and 14 days post inoculation; dpi)], but experiments for different viruses were carried out on separate occasions. At least 60 plants were grown for each genotype, treatment and time point. Each tray contained 60 plants (20 of each genotype) and was regularly repositioned within the growth cabinet to exclude positional effects on plant growth. Each treatment, genotype and time point had three biological replicates (total of 72 biological samples). Each biological replicate contained a pooled sample of 20 plants each.

### Virus Inoculation

The TuMV-QLD1b isolate used in this study was a serially passaged isolate of an original sample (VIR-0745; TuMV-QLD1a) previously sourced from the Queensland Department of Agriculture and Fisheries (DAF) in 2007 (Pretorius et al., 2016). Similarly, CaMV- Dar78694 and the AltMV virus isolates were also supplied from the DAF collection (Pretorius et al., 2017). CMV isolate K was kindly supplied by John Randle in 2004 (Moyle et al., 2018). *Nicotiana benthamiana* plants were used to propagate TuMV and AltMV, while tomato wild type (Moneymaker) was used to propagate CMV. *Brassica rapa* subsp. *chinensis* leaves were inoculated with CaMV and used for propagation. All virus inoculations of the wild-type *Arabidopsis* plants were performed by fresh inoculums. A 100 mM sodium phosphate buffer, pH 7.4 containing 1 g/L sodium sulphite was used to suspend the leaves after crushing. Abrasion was done by using celite. The following steps included gently rubbing three leaves per plant and then leaving the inoculum on the leaves for about 5 min before washing it off. For controls, mock inoculations were performed using the same buffer and abrasive.

### RNA Extraction and cDNA Synthesis

Two time points (2, 14 dpi) were used to collect the *Arabidopsis* plants. The foliar parts of the plants were used for analysis after cutting the plants at the base. Then, these parts were dropped into liquid nitrogen immediately including three replicates with 20 pooled plants per replicate (combined from two different trays each), for each genotype and time point. This was followed by grounding the tissue samples in liquid nitrogen. RNA was extracted using the Promega SV Total RNA Isolation System by following the manufacturer’s instructions. A Nanodrop spectrophotometer (Thermo Scientific, Australasia) was used to measure the concentrations of the purified RNA samples. Agarose gel electrophoresis was used to confirm the intact quality of the RNA samples. cDNA was generated by using components of the Tetro cDNA synthesis kit (BIOLINE, Australasia), including 1 µl reverse transcriptase for 2.5 µg of total RNA in 20 µl reactions containing 0.5 µl random hexamers, 0.5 µl oligo dT primers, 1 µl 10 mM dNTP mix, 4 µl 5x RT Buffer, and 1 µl RNase Inhibitor with the following thermocycling program 25°C for 10 min, 45°C for 30 min. The reaction was finished by incubating at 85°C for 5 min.

### DNA Extraction

Extraction of plant DNA was performed by crushing plant material with a mortar and pestle in liquid nitrogen, then subjecting it to the CTAB method to extract DNA (Graham et al., 1994).

### Real-Time Quantitative Reverse Transcriptase PCR

The ViiA 7 Real-Time PCR system with SYBR green was used to perform real-time quantitative PCR (qPCR) to measure the virus titers of CaMV or real-time quantitative reverse transcriptase PCR (qRT-PCR) to measure virus titers of TuMV, AltMV, CMV, or gene transcript abundances with three technical replicates per run. Primer sequences are shown in **Supplementary Table 1**. Primer Express software was used to quantify the abundance of virus sequences or gene transcripts relative to the housekeeping reference genes *β-ACTIN2*, *β-ACTIN7*, and *β-ACTIN8*. qRT-PCR assays were performed in a 10 µl reaction containing 5 µl SYBR Green Master MIX, 1 µl of primer mix (0.3 µM for each primer), and 4 µl of cDNA templates which were diluted 12.5 times prior to PCR reactions. The PCR conditions were as follows: 95°C for 10 min, 40 cycles for 15 s at 95°C, 60°C for 1 min. The melting curve conditions were 95°C for 15 s, 60°C for 1 min, and 95°C for 15 s. LinRegPCR™ software was used based on the following equation:

Relative abundance=(Egene˄(−Ct gene)/(E ACTIN˄(−Ct ACTIN)

as previously described (Liu et al., 2016; Fallath et al., 2017).

Technical replicates where the higher value had more than 0.5 Ct difference to the other values were excluded from the analysis as this could be due to reaction inhibition. The change of the gene expression levels was determined by taking the average of the three biological replicates and comparing that to the control plants (either wild-type virus-inoculated or mock-inoculated plants). Statistically significant differences (*P* < 0.05) were determined using Student’s t-test for pairwise comparisons or ANOVA F test followed LSD analysis for multiple comparisons. Experiments for different viruses were carried out at different times. For this reason, all comparisons to different viruses are done on the basis on whether a gene was induced or repressed within the experiments (i.e., pairwise comparisons to mock-inoculated controls or to wild type), but not absolute levels of gene expression were used.

## Results

Wild-type *Col*-0, *med18*, and *med25* mutants of *Arabidopsis thaliana* were inoculated with TuMV, CaMV, AltMV, and CMV, to test for host defense marker gene expression and virus disease progression, and to compare these across different viruses. First the phenotypes of infected plants were determined, then the virus titers were quantified by qRT-PCR (TuMV, AltMV, CMV) or qPCR (CaMV) at both, 2 dpi and 14 dpi, followed by qRT-PCR analyses for host marker gene expression at 2 dpi to gain an understanding of early defense responses that may have influenced virus resistance.

### *med18* Mutants Display Broad RNA Virus Resistance While *med25* Mutants are More Susceptible to RNA and DNA Viruses

**Figure 1:**
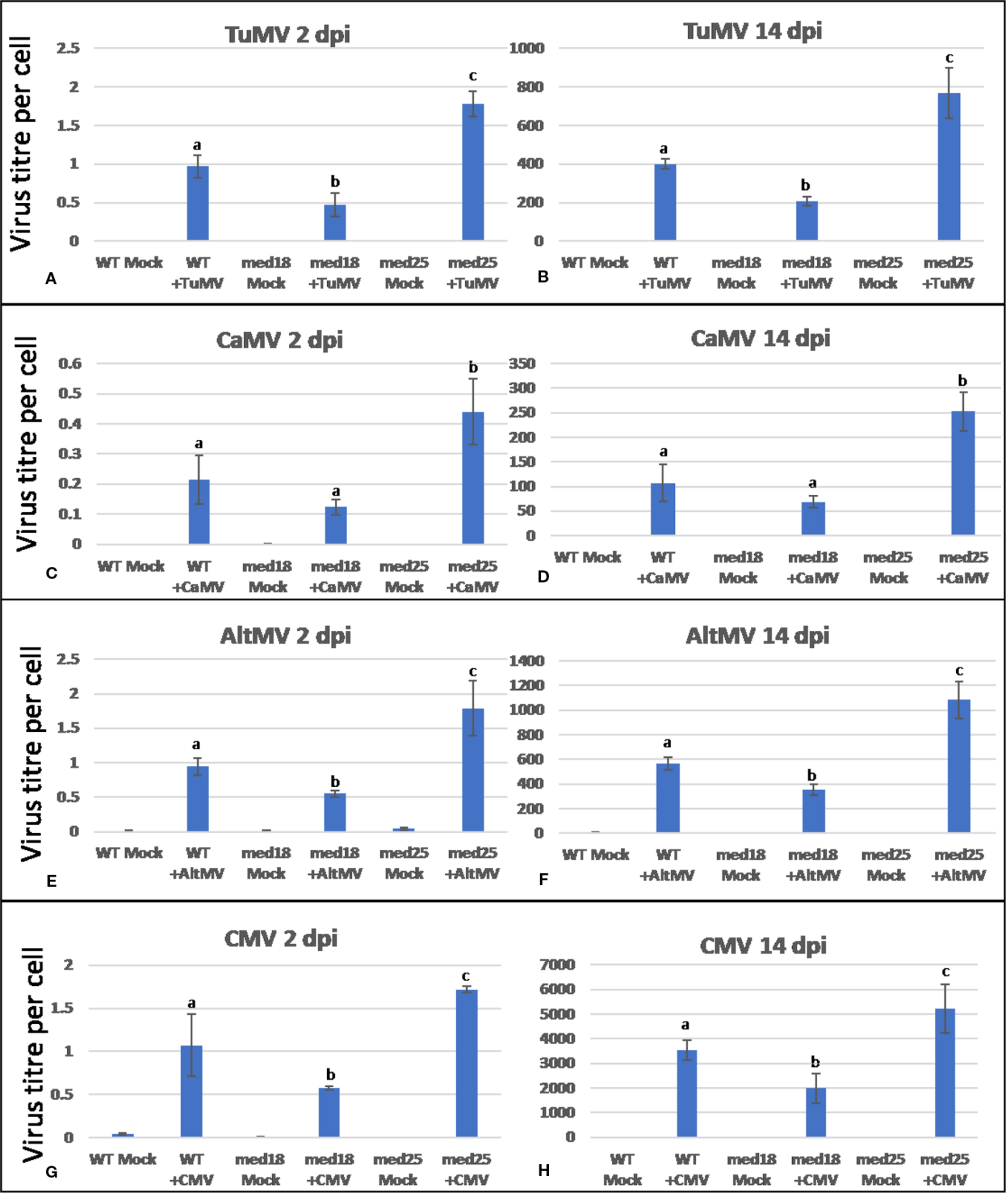
Virus titer per plant cell of *Arabidopsis* wild-type (*Col*-0), *med18*, and *med25* mutant plants inoculated with *Turnip mosaic virus* (TuMV) **(A, B)**, *Cauliflower mosaic virus* (CaMV) **(C, D)**, *Alternanthera mosaic virus* (AltMV) **(E, F)**, or *Cucumber mosaic virus* (CMV) **(G, H)** at 2 dpi and 14 dpi. Shown on the *Y* axes are quantitative reverse transcriptase PCR (qRT-PCR) or quantitative PCR (qPCR) mean values of virus abundance ± SE relative to plant cells (*ACTIN*). Different small letters indicate statistically significant (*P* < 0.05) differences between genotypes of infected plants for each time point and virus.

Inoculation of wild-type *med18* and *med25* with TuMV, CaMV, AltMV, or CMV resulted in symptom development for all viruses tested (**Supplementary Figure 1**). However, it was noted that symptoms were generally less pronounced in *med18* plants when compared to wild-type plants. On the contrary, *med25* plants typically displayed more severe symptoms. Virus titer quantification by qRT-PCR confirmed that indeed, all *med18* plants harbored significantly (*P* < 0.05) less RNA viruses than wild-type plants at an early stage (2 dpi) as well as at a more advanced state (14 dpi) of disease progression, and CaMV-infected plants also showed the same trend (**Figure 1**). However, *med25* plants consistently contained significantly (*P* < 0.05) more viruses (RNA and DNA viruses) than wild-type plants at both time points. The following sections will present data for each virus and compare these with marker gene expression in all three genotypes tested.

### TuMV’s Suppression of SA Signaling and Induction of JA Signaling Is Reversed in *med18* and *med25* Mutant Plants

**Figure 2:**
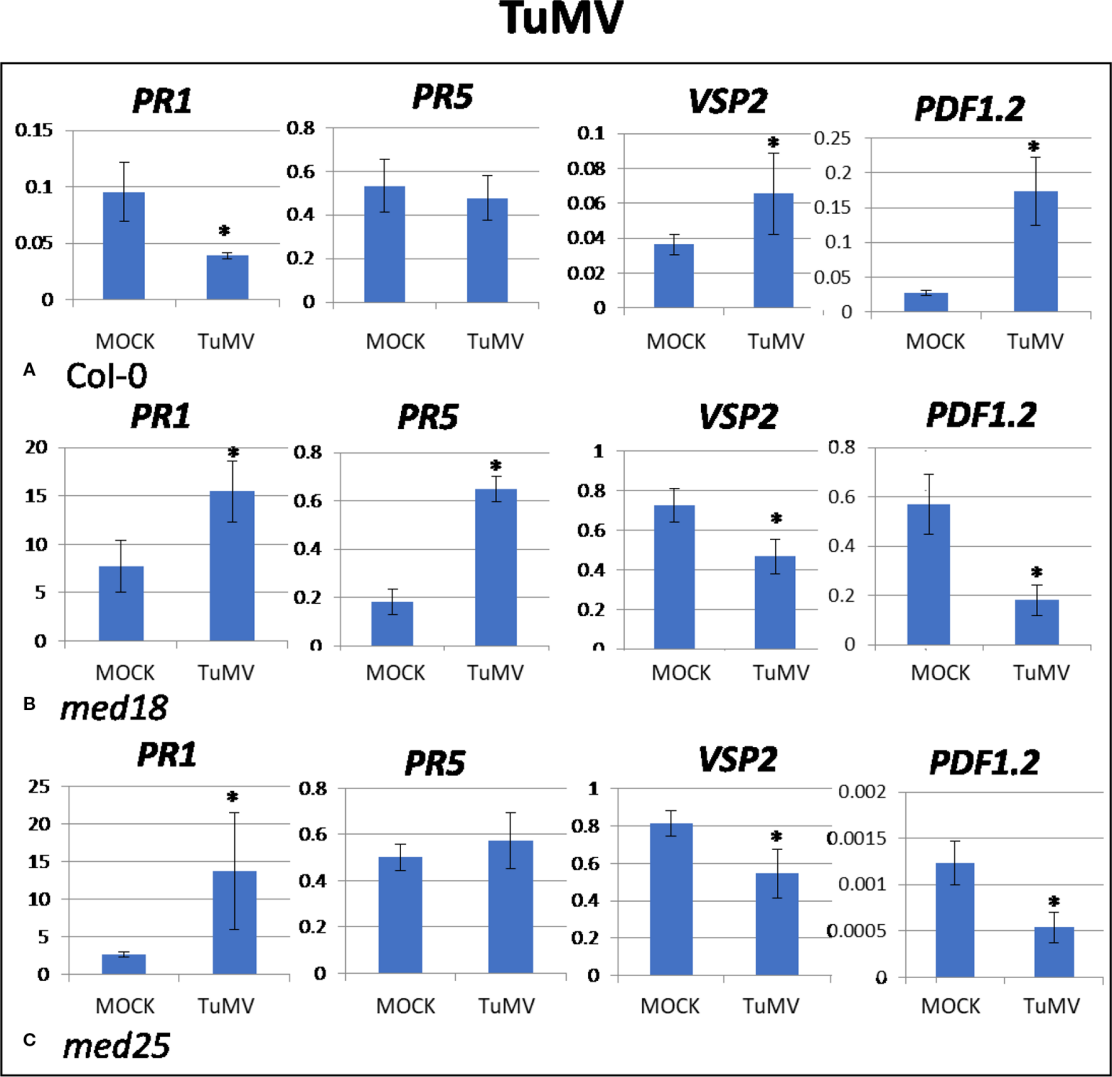
Quantitative reverse transcriptase PCR (qRT-PCR) assays with SA- (*PR1, PR5*) and JA- (*PDF1.2, VSP2*) signaling marker genes of *Arabidopsis* plants inoculated with TuMV. Shown on the *Y* axes are mean ± SE values of transcript abundances relative to housekeeping *ACTIN* genes for **(A)** wild-type *Col*-0, **(B)** *med18*, and **(C)** *med25* plants at 2 days after inoculation. Asterisks indicate significant (*P* < 0.05) induction or repression of each gene by comparing the means of mock-inoculated to TuMV-infected samples.

TuMV-inoculated plants showed clear growth reduction symptoms compared to mock-inoculated controls (**Supplementary Figure 1A**). Visual symptoms were strongest for *med25* and weakest for *med18*. The virus titer of TuMV-inoculated wild-type *Col*-0 plants, as measured by qRT-PCR, increased from approx. 1 virus per *ACTIN* transcript at 2 dpi to 400 at 14 dpi (**Figures 1A, B**). qRT-PCR *Col-*0 gene expression data at 2 dpi showed that SA signaling marker gene expression of *PR1* was reduced in the presence of TuMV, while marker genes for JA signaling (*VSP2, PDF1.2*) were upregulated (**Figure 2**). The virus titer of TuMV-inoculated *med18* plants, as measured by qRT-PCR, increased from 0.48 viruses per *ACTIN* transcript at 2 dpi to 207 at 14 dpi (**Figures 1A, B**), suggesting that *med18* plants are more resistant to TuMV than wild-type plants. The virus titer of TuMV-inoculated *med25* plants, as measured by qRT-PCR, increased from 1.78 viruses per *ACTIN* transcript at 2 dpi to 769 at 14 dpi (**Figures 1A, B**), suggesting that *med25* plants are more susceptible to TuMV than wild-type plants. In contrast to wild type, qRT-PCR gene expression data for both *med18* and *med25* plants, showed that marker genes for JA signaling (*VSP2, PDF1.2*) were downregulated, while SA marker gene expression of *PR1* was significantly increased (and *PR5* also in *med18*) in the presence of TuMV at 2 dpi (**Figure 2**). In *med25*, there was a trend to increased expression that was not significant with the number of experiments and replicates used.

### CaMV Infection Induced SA Signaling, but JA Signaling Was Suppressed in *med18* and Induced in *med25* Plants

**Figure 3:**
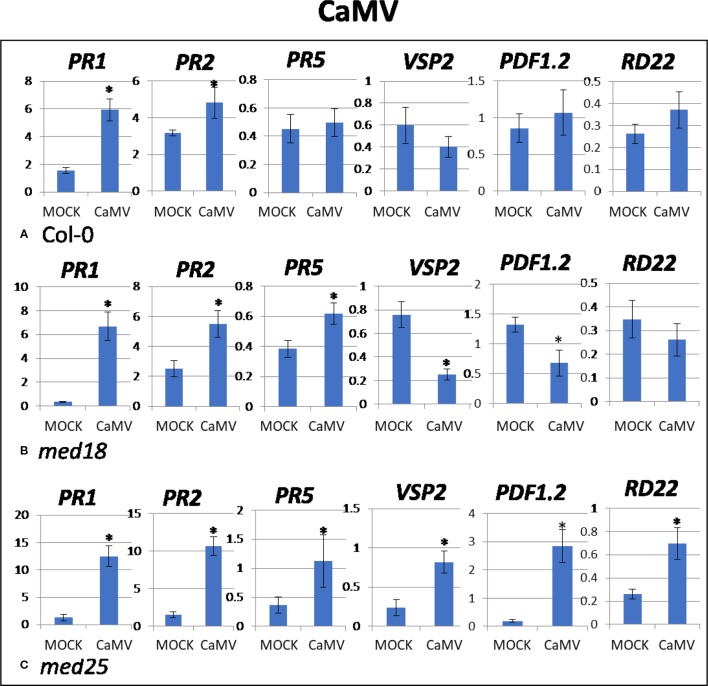
Quantitative reverse transcriptase PCR (qRT-PCR) assays with SA- (*PR1, PR5*), JA- (*PDF1.2, VSP2*) and ABA- (*RD22*) signaling marker genes of *Arabidopsis* plants inoculated with CaMV. Shown on the *Y* axes are mean ± SE values of transcript abundances relative to *ACTIN* housekeeping genes for **(A)** wild-type *Col*-0, **(B)** *med18*, and **(C)** *med25* plants at 2 days after inoculation. Asterisks indicate significant (*P* < 0.05) induction or repression of each gene by comparing the means of mock-inoculated to CaMV-infected samples.

As shown in **Supplementary Figure 1B**, CaMV-inoculated plants showed clear growth reduction symptoms compared to mock-inoculated controls. Visual symptoms were strongest for *med25* and weakest for *med18*. The virus titer of CaMV-inoculated wild-type plants, as measured by qPCR, increased from 0.21 viruses per cell at 2 dpi to 107 at 14 dpi (**Figures 1C, D**). qRT-PCR gene expression data showed that marker genes for JA signaling (*VSP2, PDF1.2*) were not differentially expressed, while SA marker gene expression (*PR1*) was induced in the presence of CaMV at 2 dpi (**Figure 3**). Base levels for *PR1* differed for the different replicates and also across other virus experiments, but each replicate showed clear induction for *PR1* following CaMV infection. To more broadly profile gene expression for SA signaling, but also for ABA signaling, *PR2* (SA signaling), and *RD22* (ABA signaling) were included in the analysis. Both of these genes also showed upregulation in the presence of CaMV at 2 dpi (**Figure 3**).

The virus titer of CaMV-inoculated *med18* plants, as measured by qPCR, increased from 0.12 viruses per cell at 2 dpi to 69 at 14 dpi (**Figures 1C, D**). Although there was a trend for reduced CaMV accumulation in *med18* plants, it was not significantly different from the wild-type plants at either 2 or 14 dpi. Similar to wild-type plants, qRT-PCR gene expression data from *med18* plants in the presence of CaMV at 2 dpi showed that marker genes for SA signaling (*PR1, PR2,* and the trend for *PR5*) were upregulated, while JA signaling (*VSP2, PDF1.2*) was downregulated (**Figure 3**). The virus titer of CaMV-inoculated *med25* plants, as measured by qPCR, increased from 0.44 viruses per cell at 2 dpi to 253 at 14 dpi (**Figures 1C, D**), indicating that *med25* plants are more susceptible to CaMV compared to wild type. qRT-PCR gene expression data showed that marker genes for SA-, JA- and ABA- (*RD22*) signaling were all upregulated in the presence of CaMV at 2 dpi (**Figure 3**).

### AltMV Infection Induced SA and JA Signaling, but JA Signaling Was Suppressed in *med18* Plants

**Figure 4:**
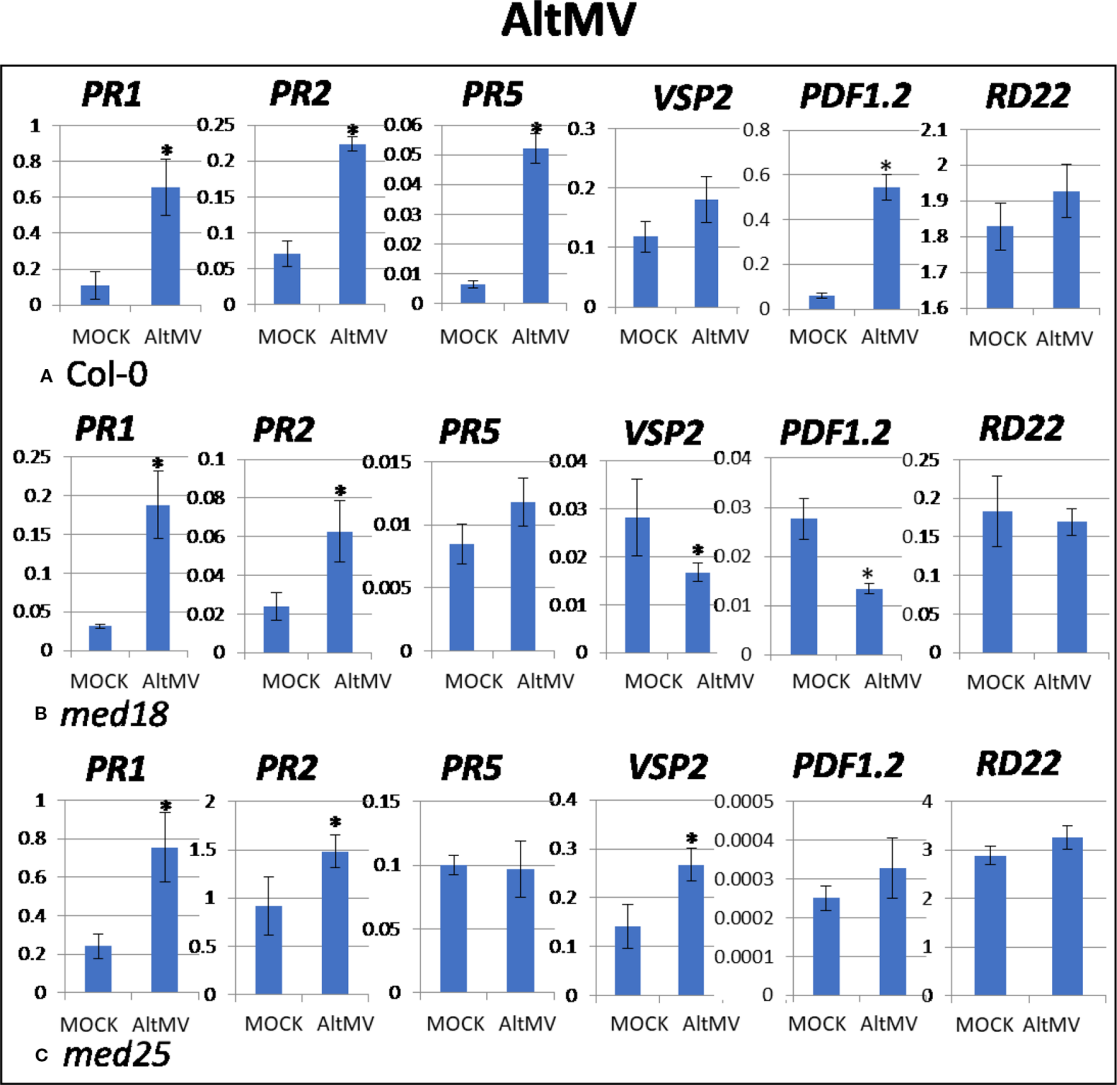
Quantitative reverse transcriptase PCR (qRT-PCR) assays with SA- (*PR1, PR5*), JA- (*PDF1.2, VSP2*), and ABA- (*RD22*) signaling marker genes of *Arabidopsis* plants inoculated with AltMV. Shown on the *Y* axes are mean ± SE values of transcript abundances relative to *ACTIN* housekeeping genes for **(A)** wild-type *Col*-0, **(B)** *med18*, and **(C)** *med25* plants at 2 days after inoculation. Asterisks indicate significant (*P* < 0.05) induction or repression of each gene by comparing the means of mock-inoculated to *Alternanthera mosaic virus* (AltMV)–infected samples.

As shown in **Supplementary Figure 1C**, AltMV-inoculated plants showed clear growth reduction symptoms compared to mock-inoculated controls. Visual symptoms (based on biomass) across different genotypes were similar. However, the virus titer of AltMV-inoculated wild-type plants, as measured by qRT-PCR, increased from 0.94 viruses per *ACTIN* transcript at 2 dpi to 567 at 14 dpi (**Figures 1E, F**). qRT-PCR gene expression data showed that marker genes for JA signaling (*PDF1.2*) and SA signaling (*PR1, PR2, PR5*) were upregulated after infection with AltMV at 2 dpi, and a second JA signaling pathway gene (*VSP2*) showed the same trend (**Figure 4**).

The virus titer of AltMV-inoculated *med18* plants, as measured by qRT-PCR, increased from 0.55 viruses per *ACTIN* transcript at 2 dpi to 355 at 14 dpi (**Figures 1E, F**), indicating that *med18* plants are more resistant compared to wild type. Similar to wild type, SA signaling (*PR1*, *PR*,*2* and the trend for *PR5*) was upregulated in *med18* after infection. Interestingly in contrast to wild-type and *med25* plants, qRT-PCR gene expression data showed that marker genes for JA signaling (*VSP2, PDF1.2*) were downregulated in the presence of AltMV in *med18* at 2 dpi (**Figure 4**).

For *med25* AltMV-inoculated plants, the virus titer, as measured by qRT-PCR, increased from 1.79 viruses per *ACTIN* transcript at 2 dpi to 1,083 at 14 dpi (**Figures 1E, F**), indicating that *med25* plants are more susceptible compared to wild type. qRT-PCR gene expression data showed that marker genes for SA signaling (*PR1, PR2,* but not *PR5*) and JA signaling (*VSP2*) were upregulated in *med25* in the presence of AltMV at 2 dpi (**Figure 4**). ABA pathway marker gene *RD22* was not significantly differentially expressed following AltMV infection for any time point or genotype.

### CMV Infection Induced *PR1* Involved in SA Signaling and JA Signaling was Further Suppressed in *med18* and *med25* Plants

**Figure 5:**
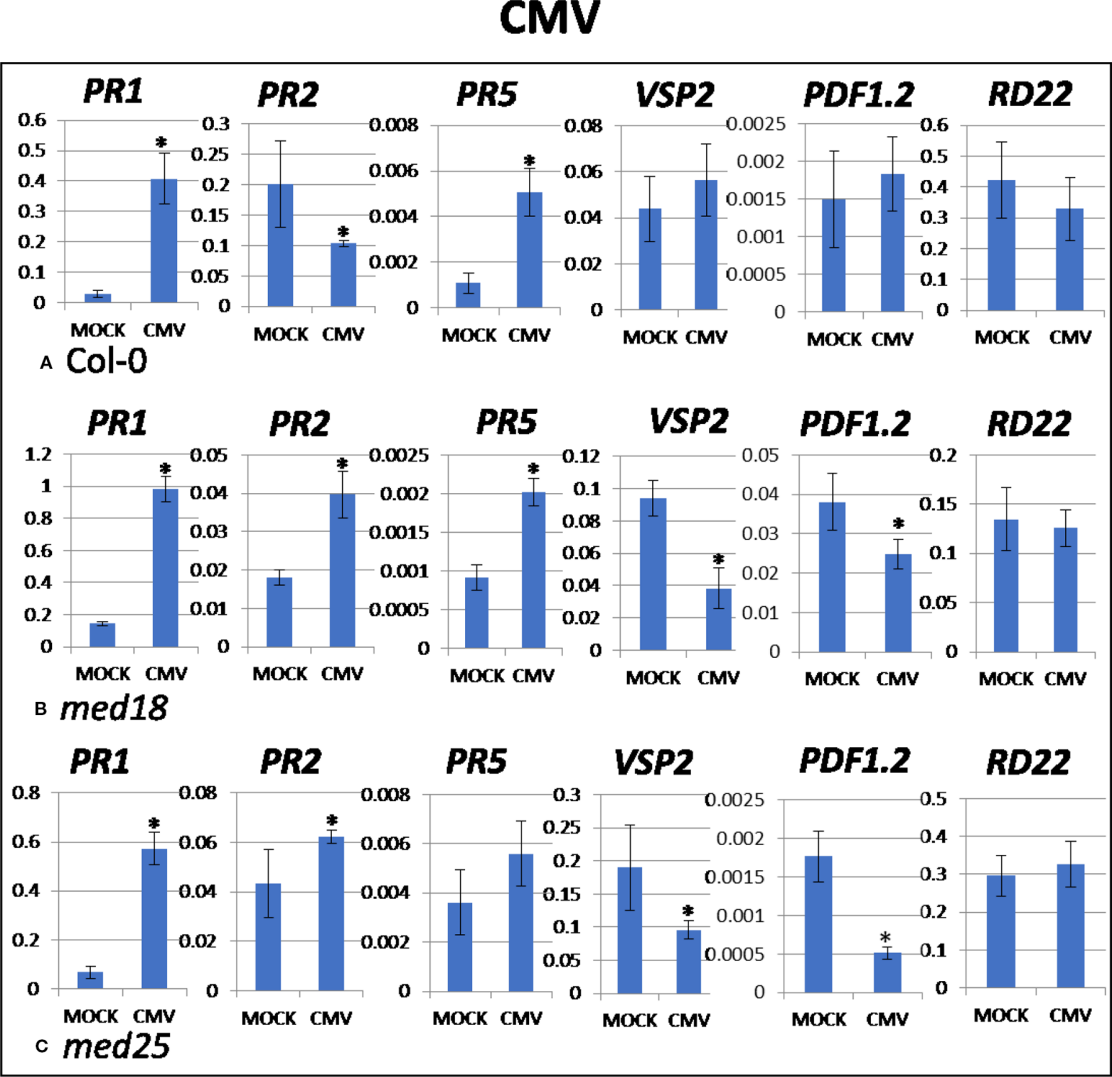
Quantitative reverse transcriptase PCR (qRT-PCR) assays with SA- (*PR1, PR5*), JA- (*PDF1.2, VSP2*), and ABA- (*RD22*) signaling marker genes of *Arabidopsis* plants inoculated with CMV. Shown on the *Y* axes are mean ± SE values of transcript abundances relative to *ACTIN* housekeeping genes for wild-type **(A)** *Col*-0, **(B)** *med18*, and **(C)** *med25* plants at 2 days after inoculation. Asterisks indicate significant (*P* < 0.05) induction or repression of each gene by comparing the means of mock-inoculated to CMV-infected samples.

As shown in **Supplementary Figure 1D**, CMV-inoculated plants showed clear growth reduction symptoms compared to mock-inoculated controls. Visual symptoms were strongest for *med25* and weakest for *med18*. The virus titer of CMV-inoculated wild-type plants, as measured by qRT-PCR, increased from 1.07 viruses per *ACTIN* transcript at 2 dpi to 3,544 at 14 dpi (**Figures 1G, H**). qRT-PCR expression data showed that genes for SA signaling (*PR1, PR5* but not *PR2*) were upregulated in the presence of CMV at 2 dpi (**Figure 5**). There was no significant change in JA-signaling markers on CMV infection of *Col*-0 plants.

The virus titer of CMV-inoculated *med18* plants, as measured by qRT-PCR, increased from 0.57 viruses per *ACTIN* transcript at 2 dpi to 1994 at 14 dpi (**Figures 1G, H**), indicating that *med18* mutants are more resistant than wild-type plants. qRT-PCR expression data showed that SA signaling (*PR1, PR2, PR5*) was upregulated, while a gene for JA signaling (*VSP2*) was downregulated in the presence of CMV at 2 dpi (**Figure 5**). The virus titer of CMV-inoculated *med25* plants increased from 1.72 viruses per *ACTIN* transcript at 2 dpi to 5218 at 14 dpi (**Figures 1G, H**), indicating that *med25* mutants are more susceptible than wild-type plants. qRT-PCR expression data showed that SA signaling (*PR1*, *PR2*, but not *PR5*) was upregulated, while genes for JA signaling (*VSP2, PDF1.2*) were downregulated in the presence of CMV at 2 dpi (**Figure 5**). ABA pathway marker gene *RD22* was not significantly differentially expressed following CMV infection for any time point or genotype.

## Discussion

Studies on TuMV, CaMV, AltMV, and CMV are crucial in providing a better understanding of the impact that these viruses have on *Arabidopsis*. Many studies have attempted to elucidate the mechanism by which the virus affects plants leading to systemic infections (Zvereva and Pooggin, 2012; Revers and Nicaise, 2014; Roossinck, 2015). Results obtained in the present work suggest that the mechanisms vary widely for different viruses in *Arabidopsis*. For example, while SA signaling was activated upon CaMV, AltMV, and CMV inoculation of wild-type plants at 2 dpi (**Figures 3A**, **4A**, and **5A**), it was downregulated when plants were inoculated with TuMV (**Figure 2A**). JA signaling was also upregulated by TuMV and AltMV, but not CaMV and CMV. It should be emphasized that all viruses used in this study are compatible and are able to infect and proliferate in *Arabidopsis* plants (**Figure 1**; **Supplementary Figure 1**). Therefore, these viruses, and unlike their incompatible counterparts, have evolved to effectively evade the plant’s defense system. This can be achieved in different ways, but one of the strategies virulent plant pathogens can use, is to “hijack” a defense pathway that makes plants more susceptible. Wild-type plants infected with TuMV showed reduced SA and increased JA signaling (**Figure 2**), suggesting that TuMV may be able to hijack the JA pathway to make plants more susceptible (e.g. by counteracting SA signaling and oxidative burst). This phenomenon has been found for several plant pathogens, in particular *F. oxysporum* and *P. syringae* (Edgar et al., 2006; Kidd et al., 2009; Thatcher et al., 2009). Both are hemibiotrophic pathogens where early SA signaling/oxidative burst would be effective to fight the pathogens in their initial biotrophic phase. However, instead, JA signaling is upregulated which suppresses oxidative burst and SA signaling, presumably making plants more susceptible.

Many other mechanisms also play a role in viral disease progression, in particular the role of viral genes targeted to the nucleus and RNAi pathways should be considered. TuMV’s viral-genome-related protein of the nuclear inclusion protein interrelates with the potyviral VP-g interrelating protein that has a role in the transcriptional control and this interrelation might interfere with the host gene expression (Dunoyer et al., 2004). In addition, TuMV also has another nonstructural protein that tends to accrue in the nucleus and has been known to inhibit RNA silencing, assistance component proteinase (Mallory et al., 2002; Kasschau et al., 2003) that is comparable to CMV 2b that is described as a strong inhibitor of RNA silencing and RNA-directed DNA methylation (Wang et al., 2004). By comparison, AltMV protein gene block 1 is based in the nucleus and is also labeled as a suppressor of RNA silencing (Nam et al., 2013). Finally, CaMV even though a DNA virus, also duplicates in the cytoplasm through RNA intermediates. Moreover, its protein 6 is also known to accrue in the nucleus with one of its responsibility being to suppress RNA muzzling by interacting with the host nuclear protein DRB4 (Love et al., 2012). All viruses used in this research have the potential to interfere with the RNAi pathway and this should be the subject of further investigations.

Two Mediator subunits, MED18 and MED25, have been investigated in the present study with the aim to better understand their function in defense signaling and to establish their potential role in plant virus resistance. Both subunits play an established role in JA signaling, but are located at opposite ends of the Mediator complex (Kidd et al., 2009; Fallath et al., 2017). Mutants of both subunits show resistance against the root-infecting hemibiotrophic pathogen *F. oxysporum*, but are more susceptible to necrotrophic leaf pathogens *B. cinerea* and *A. brassicicola* (Kidd et al., 2009; Çevik et al., 2012; Fallath et al, 2017). This made these two subunits particularly interesting to study for virus resistance and defense against viruses that are obligate biotrophs and we hypothesized that the corresponding mutants may show an altered phenotype toward viral resistance. Indeed, *med25* mutants were consistently more susceptible to all viruses tested, whereas *med18* plants were consistently more resistant when compared to wild type. This was observed for both, 2 dpi and 14 dpi (**Figure 1**).

*med18* plants reportedly show down-regulation of JA signaling defense and biosynthesis genes, while SA-associated PR-, ROS producing and scavenging genes are upregulated (Fallath et al, 2017). It is likely that the enhanced SA signaling capability in these plants led to a more pronounced HR resulting in faster oxidative burst and the production of ROS, leading to programmed cell death of virus-infected cells and therefore limiting spreading of viruses in the plants. Indeed, the gene expression analysis of virus-infected *med18* plants consistently demonstrated that JA marker genes were downregulated, and SA genes were upregulated at 2 dpi, independent of which virus infected the plants (**Figures 2**–**5**). Similarly, JA signaling genes were suppressed under *F. oxysporum* infection in *med18*, while *PR1* and *PR5*, as well as several genes linked to ROS production were upregulated (Fallath et al., 2017). *med18* plants are more sensitive to SA signaling, but there were differences for different SA marker genes. After SA treatment, *PR1* and to a lesser extend *PR5* (but not *PR2*) expression was higher in *med18* compared to wild type, indicating that MED18 differentially regulates various genes of the SA pathway in a different manner. The present study found that various viruses modulate SA signaling in a different manner in wild-type and *med18* mutants. For example, *PR1* was higher expressed in *med18* upon TuMV and CMV infection but less expressed during AltMV infection, while *PR5* was upregulated in AltMV- and CMV-infected *med18* compared to wild type.

JA signaling and singlet oxygen stress signaling could be controlled through the Mediator complex *via* MED18, or otherwise, faulty JA signaling in *med18* leads to increased ROS production and tolerance. No major lesions could be observed in virus-infected *med18* plants upon microscopic observations, suggesting that the spread of viruses is similarly restricted as previously reported for *F. oxysporum* in *med18* (Fallath et al., 2017). It was also shown that MED18 is recruited to the promoter of *WRKY33* leading to increased *WRKY33* expression (Liao et al., 2016). Similar to *MED18*, the inactivation of *WRKY33* results in reduced JA and increased SA defense responses and higher susceptibility to *B. cinerea* (Liu et al., 2017) and therefore the mechanism of the MED18-controlled JA/SA crosstalk found in the present study could be based on MED18’s recruitment to the WRKY33 promoter. MED18 has also been reported to interact with TFs, YIN YANG1, ABA INSENSITIVE4 and SUPPRESSOR OF FRIGIDA4 (Lai et al., 2014), and future research could test whether these TFs also affect virus resistance.

*med25* mutants, on the other hand, are only partially insensitive to JA but also show a reduction in JA-associated gene expression that was linked to the *F. oxysporum* resistance phenotype, although to a lesser extent than *med18* plants (Kidd et al., 2009; Fallath et al., 2017). Interestingly, *med25*, showed increased susceptibility to all viruses tested (**Figure 1**), suggesting that this Tail-located subunit plays a role in antivirus defense signaling (rather than normal virus infection). This may include the RNAi pathway. As explained by Kim et al. (2011), MED25, MED18, and MED20a all play a role in the microRNA biogenesis pathway. Indeed, it was found that the subunits were engaged in encouraging the transcription of miRNA genes and were also in charge of recruiting RNA-directed RNA Polymerase 2 (RDR2) to certain promoters. In addition, the Mediator complex is involved when it comes to transcriptional gene silencing that is regulated by small interfering RNAs (Kim et al., 2011). The intricacy of the Mediator complex as well as the initiation of RDR2 transcription has been equated to an ancient type of cellular defense in averting DNA and RNA factors like transposons and viruses from attacking the host transcriptional machinery (Madhani, 2013). This is likely to expound the reason why Mediator subunits might be targeted by viruses as has previously been shown for the herpes simplex virus VP16 interaction domain (Bäckström et al., 2007; Aguilar et al, 2014). Plant viral interference with Mediator subunits would effectively modulate their roles in mRNA transcription, miRNA gene transcription, and transcriptional gene silencing.

## Conclusion

The results obtained from this study provide a more comprehensive understanding of antiviral resistance in plants, progression of viral infections and the role of Mediator subunits in this process. The role of two Mediator subunits, MED18 and MED25, required for normal JA signaling, was tested, but that are located at the Head and Tail domain of the Mediator complex, respectively. Infection with all viruses exhibited clear growth reduction in plants, with visible symptoms being strongest in *med25* mutants and weakest in *med18* mutants. All wild-type plants showed an upregulation of SA signaling when infected with all viruses except TuMV which showed a downregulation in SA signaling and upregulation in JA signaling. This suggests that TuMV possesses a mechanism capable of increasing the plant’s susceptibility to infection. On infection with TuMV, CaMV, CMV, or AltMV, *med25* plants were found to be susceptible to infection, while *med18* plants were found to be resistant against all viruses tested. *med18*’s resistance could be explained by the finding that virus-infected *med18* plants had JA marker genes downregulated, while SA genes were upregulated at 2 dpi, independent of which virus infected the plants. The results obtained from this study confirmed our hypothesis that *med18* mutants would be resistant to viral infection based on their defective JA and increased SA signaling, therefore establishing a firm role of MED18 for normal virus infection. In contrast, *med25* mutants were susceptible to all viral infections which suggest that MED25-mediated gene expression is required for plant defense signaling against viruses and further studies may reveal whether MED25 is also needed to interfere with viral mechanisms that inhibit RNA silencing.

## Data Availability Statement

The datasets generated for this study are available on request to the corresponding author.

## Author Contributions

NH performed most experiments and contributed to manuscript writing and data interpretation. LS contributed to experimental design and data interpretation. EL performed experiments and interpreted data. JB performed experiments and interpreted data. EA performed experiments and interpreted data. SD contributed to manuscript writing. PS contributed to experimental design, data interpretation and manuscript writing.

## Funding

We wish to thank the Iraqi Ministry of Higher Education and Iraqi Cultural Attaché for financial support and for kindly providing a scholarship to the first author Nasser Kadhum Hussein).

## Conflict of Interest

The authors declare that the research was conducted in the absence of any commercial or financial relationships that could be construed as a potential conflict of interest.
