# Supplementary material for: Suppression of Arabidopsis Mediator Subunit-Encoding MED18 Confers Broad Resistance Against DNA and RNA Viruses While MED25 Is Required for Virus Defense
Source: Front Plant Sci. 2020 Mar 4;11:162. doi: 10.3389/fpls.2020.00162 (PMC7064720; doi:10.3389/fpls.2020.00162)
Supplement: Supplementary file 1 [file Image_1.pdf]

A-TuMV

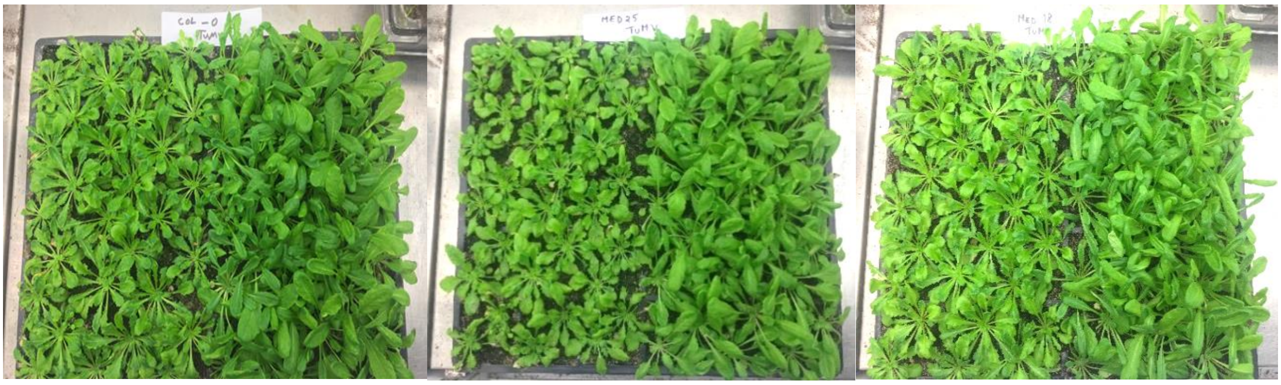

Col-0 *med25* *med18*

B-CaMV

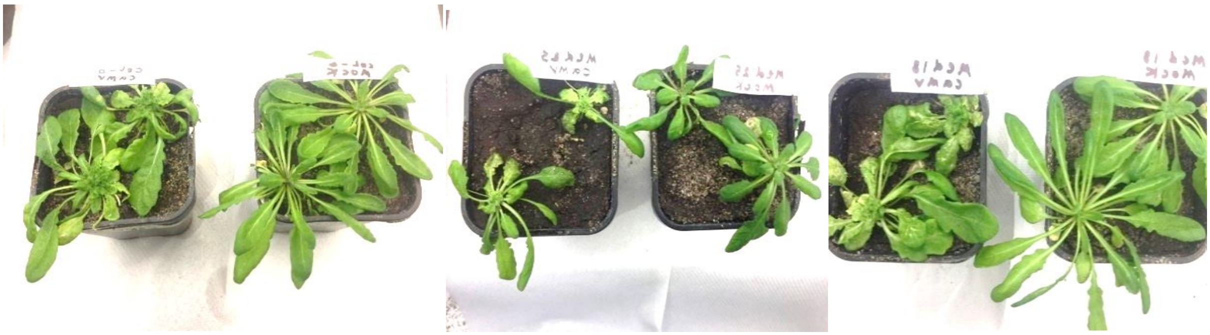

Col-0 *med25* *med18*

C-AltMV

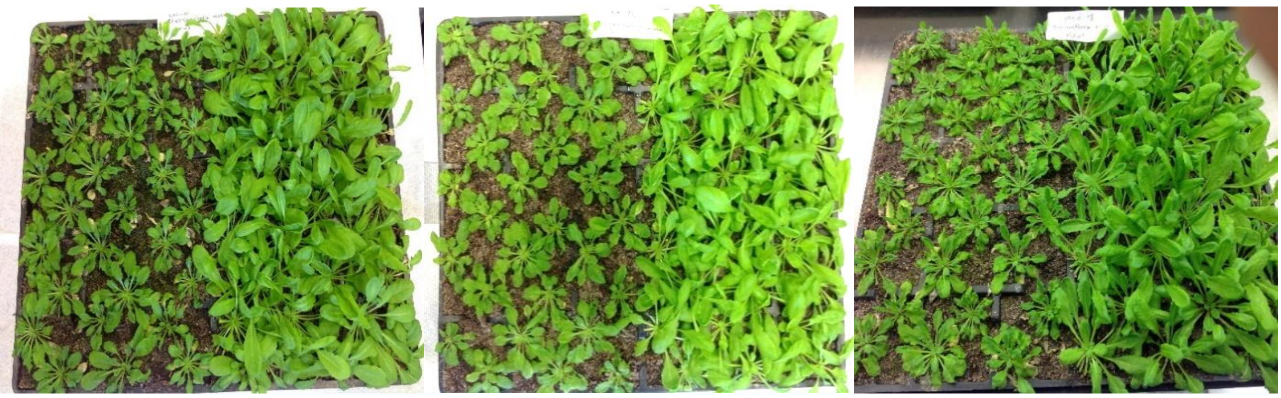

Col-0 *med25* *med18*

D-CMV

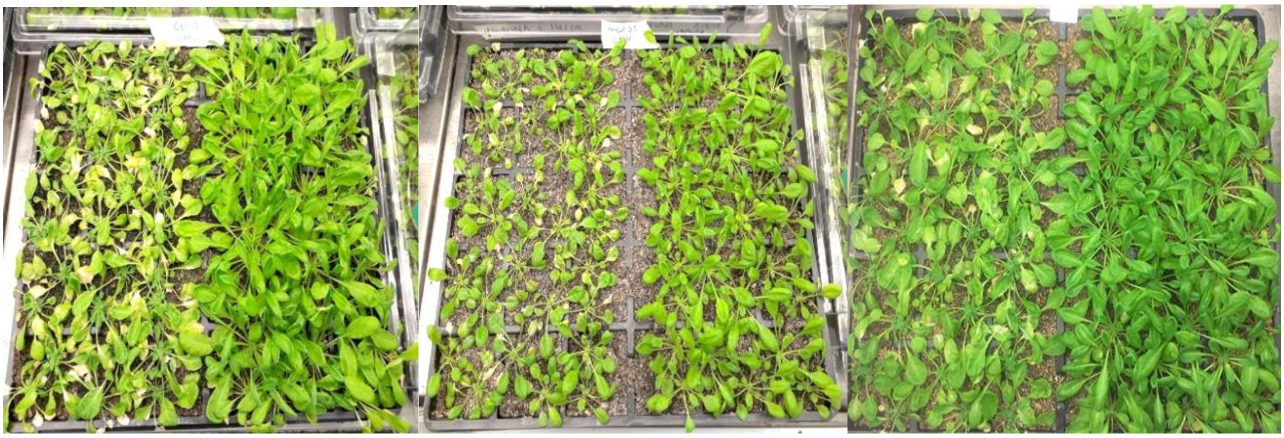

Col-0 *med25* *med18*

Supplementary Figure 1. Virus-infected *Arabidopsis thaliana* (*Col*-0), *med25* and *med18* plants. **A**-TuMV (14 dpi), **B**-CaMV (21 dpi), **C-** AltMV (14 dpi), **D**-CMV (14 dpi). Shown for each are virus-inoculated (left) vs mock-inoculated (right) plants.
