# Supplementary material for: Suppression of Arabidopsis Mediator Subunit-Encoding MED18 Confers Broad Resistance Against DNA and RNA Viruses While MED25 Is Required for Virus Defense
Source: Front Plant Sci. 2020 Mar 4;11:162. doi: 10.3389/fpls.2020.00162 (PMC7064720; doi:10.3389/fpls.2020.00162)
Supplement: Supplementary file 2 [file Table_1.docx]

**Supplementary Table 1.** List of primers used for real-time PCR

| **Primer name** | **Sequence (5’ to 3’)** |
| --- | --- |
| rt_Actin 8_r | GAGGATAGCATGTGGAACTGAGAA |
| rt_Actin 7_r | GAGGAAGAGCATTCCCCTCGTA |
| rt_Actin Uni_f | AGTGGTCGTACAACCGGTATTGT |
| rt_Actin 2_r | GATGGCATGGAGGAAGAGAGAAAC |
| RtTuMV_F | TCGAGCGTTACGGAATTTCAG |
| RtTuMV_R | GATGATCATACAGCGCTTGCA |
| AltMV_F | GGTCTCACTCTCACAGGTTGC |
| AltMV_R | GTCTTGTCAGTTCTGAGGTTCC |
| RtCaMV_F | GCCCAGTAAAAGCCCTCACA |
| RtCaMV_R | GCTTCTCGGCTTCATTGTTGA |
| Rt-CMV-KCP-A | TGATTCTACCGTGTGGGTGAC |
| Rt-CMV-KCP-B | AGTACCGGTGAGGCTCCGTC |
| PDF1.2_F | AAGTTTGCTTCCATCATCACCC |
| PDF1.2_R | ATTGCCGGTGCGTCGAAAG |
| VSP2_F | GAAAACCATCTTTGGGAACG |
| VSP2_R | CGGTTTTGGAGTCGTATTGG |
| PR1_F | CTCATACACTCTGGTGGG |
| PR1_R | ATTGCACGTGTTCGCAGC |
| PR5_F | AATGTCAAGCTGGGGA |
| PR5_R | AGGTGCTCGTTTCGTC |
| PR2_F | CTCATCCTCGACGTTCCCAG |
| PR2_R | AGAAACCGCGTTCTCGATGT |
| RD22_F | ATTGTGCGACGTCTTTGGAGT |
| RD22_R | TGCGTTCTTCTTAGCCACCTC |
